# Somatotropic Axis Dysfunction in Non-Alcoholic Fatty Liver Disease: Beneficial Hepatic and Systemic Effects of Hormone Supplementation

**DOI:** 10.3390/ijms19051339

**Published:** 2018-05-02

**Authors:** Daniel Cabrera, Claudio Cabello-Verrugio, Nancy Solís, Diego San Martín, Catalina Cofré, Margarita Pizarro, Juan Pablo Arab, Johanna Abrigo, Fabián Campos, Betzabé Irigoyen, Gonzalo Carrasco-Avino, Katiuska Bezares, Valentina Riquelme, Arnoldo Riquelme, Marco Arrese, Francisco Barrera

**Affiliations:** 1 Departament of Gastroenterology, School of Medicine, Pontificia Universidad Católica de Chile, Santiago 8320000, Chile; dacabrer@uc.cl (D.C.); nsolisl@gmail.com (N.S.); dsanmartin@msn.com (D.S.M.); catalina.cofre.m@gmail.com (C.C.); pizarro@med.puc.cl (M.P.); jparab@gmail.com (J.P.A.); a.riquelme.perez@gmail.com (A.R.); marrese@uc.cl (M.A.); 2 Faculty of Biological Sciences, Universidad Andrés Bello, Santiago 8320000, Chile; claudio.cabello@unab.cl (C.C.-V.); j.abrigo.leon@gmail.com (J.A.); fcamposaroca@gmail.com (F.C.); bet.irigoyen@gmail.com (B.I.); 3 Departament of Pathotology, Clínica Las Condes, Santiago 8320000, Chile; gcarrasco@clinicalascondes.cl; 4 Department of Pathology, Hospital Clínico Universidad de Chile, Santiago 8320000, Chile; 5 Department of Pathology, Hospital Clínico San Juan de Dios, Santiago 8320000, Chile; kat.bezares@gmail.com; 6 Faculty of Arts, Pontificia Universidad Católica de Chile, Santiago 8320000, Chile; valeriquelme.98@gmail.com; 7 Department of Health Sciences, Faculty of Medicine, Pontificia Universidad Católica de Chile, Santiago 8320000, Chile; 8 Centro de Envejecimiento y Regeneración (CARE), Pontificia Universidad Católica de Chile, Santiago 8320000, Chile

**Keywords:** fatty liver, somatotropic axis, growth hormone, insulin growth factor 1, IGF-1

## Abstract

Background: Somatotropic axis dysfunction associated with non-alcoholic fatty liver disease (NAFLD) has potential multisystemic detrimental effects. Here, we analysed the effects of growth hormone (GH) and insulin*-like* growth factor-1 (IGF-1) supplementation on liver histology, adipokine profile and muscle function in an NAFLD model. Methods: C57BL/6 mice were fed with a high fat diet (HFD) for 12 weeks and were separated into three groups treated for 4 weeks with: (1) High fat diet (HFD) (*n* = 10); (2) HFD + GH 9 μg/g/d (*n* = 10); (3) HFD + IGF-1 0.02 µg/g/d (*n* = 9). A control group fed a chow diet was included (*n* = 6). Liver histology, liver triglycerides content, serum alanine aminotransferase (ALT) activity, adiponectin and leptin serum levels, in vivo muscle strength, tetanic force and muscle fibre cross-sectional area (CSA) were measured. Results: HFD + GH and HFD + IGF-1 groups showed significantly lower ALT activity compared to HFD (*p* < 0.01). Liver triglyceride content in HFD + GH was decreased compared to HFD (*p* < 0.01). Histologic steatosis score was increased in HFD and HFD + GH group (*p* < 0.01), whereas HFD + IGF-1 presented no difference compared to the chow group (*p* = 0.3). HFD + GH group presented lower serum leptin and adiponectin levels compared to HFD. GH and IGF-1 supplementation therapy reverted HFD-induced reduction in muscle strength and CSA (sarcopenia). Conclusions: GH and IGF-1 supplementation induced significant improvement in liver steatosis, aminotransferases and sarcopenia in a diet-induced NAFLD model.

## 1. Introduction

Non-alcoholic fatty liver disease (NAFLD) is a highly prevalent disease, defined as fat accumulation in >5% of the hepatocytes, in the absence of excessive intake of alcohol or other hepatic disease [1]. NAFLD is used as an umbrella term that englobes a less aggressive disease called simple steatosis and a more aggressive form of the disease associated with inflammation and hepatocellular injury, known as non-alcoholic steatohepatitis (NASH) [2]. Simple steatosis is associated with a low risk of progression to cirrhosis (3.1% after 14 years of follow-up) while NASH is associated with a 38% risk of fibrosis development, a 9.8% risk of cirrhosis, and a 2.8% risk of mortality due to hepatic cause after 13 years follow-up [2,3]. It is estimated that 20% of NAFLD subjects present with NASH [4,5]. NAFLD prevalence has increased during the last decades, currently representing 20–30% of the general population [6,7].

Determinant factors of NAFLD progression to fibrosis, cirrhosis and hepatocellular carcinoma constitute an area of great interest for developing early biomarkers and eventually novel therapies for these subjects. Within the mechanisms involved, insulin resistance (IR) plays a central role [2,8,9]. IR is a complex phenomenon determined by genetic and environmental factors. The majority of NAFLD patients show IR at the muscular, adipose tissue and liver level. In addition, obese subjects modify the secretion profile of adipokines, increasing leptin, resistin, visfatin and cytokines and reducing adiponectin expression. These changes promote liver steatosis, inflammation, fibrosis and carcinogenesis [10,11,12,13].

Diverse studies have described somatotropic axis dysfunction in subjects with obesity, insulin resistance and NAFLD [14,15]. Preliminary data from our group showed that murine models of NAFLD based on a high fat diet (HFD) present dysfunction of this axis, with lower pituitary secretion of growth hormone (GH) and a reduced response to growth hormone releasing hormone (GHRH); and in the liver, a reduced expression of insulin-like growth factor-1 (IGF-1) at baseline and after GH stimulation. Similarly, our group and others have demonstrated decreased IGF-1 serum levels in insulin-resistant and NAFLD-patients that correlates significantly with liver lobular inflammation and fibrosis, even after adjusting by body mass index [16,17,18,19].

Subjects with a primary deficit of GH present a significantly increased incidence of NAFLD, and progression towards NASH, cirrhosis and liver related-death [20]. GH supplementation in these patients improves lipid profile, vascular dysfunction and optimizes insulin sensitivity [21,22,23]. At the liver level, GH supplementation drastically improves NASH, reducing the oxidative and inflammatory mediators like the tumour necrosis factor-α (TNFα) and the elevation of acute-phase proteins ((ultrasensitive C Reactive Protein (usPCR)) [24].

Previous studies in fatty liver models suggest that supplementation with GH could have beneficial effects, particularly in relation to liver steatosis [25,26]. However, GH is associated with increased lipolysis in adipose tissue, increased of gluconeogenesis and glycogenolysis at the liver and reduced glucose uptake at muscle determining a state of insulin resistance [27]. These effects are detrimental in subjects with fatty liver. In contrast, direct supplementation with IGF-1 has insulin sensitizing effects mainly mediated by the suppression of GHRH on a pituitary level and systemic reduction of GH levels, thus reducing its negative effects [23]. IGF-1 has been additionally demonstrated to have hepatoprotective effects in studies based on animal models of hepatic cirrhosis, including the decrease of oxidative stress, insulin resistance, hepatocellular apoptosis and fibrogenesis [26,28,29,30]. Given the metabolic differences in the effects of GH and IGF-1, it is relevant to compare the effects of supplementation with each of these hormones in NAFLD.

Somatotropic axis supplementation has been associated with diverse changes at the muscular tissue level and adipokines. The results on the anabolic effect at the muscular level are of particular interest. Sarcopenia is a trait increasingly described in relation to insulin resistance, obesity and fatty liver [31,32,33]. Hong et al. analysed 452 healthy subjects that underwent a computerized tomography exam and they found that subjects with sarcopenia (defined as the lower quartile of skeletal muscle index) presented a 5.16 greater risk of NAFLD in comparison with subjects in the highest quartile of the skeletal muscle index evaluated by dual energy X-ray absorptiometry [34]. Diverse factors participate in sarcopenia development in NAFLD including a decrease in physical activity, an increase of serum bile salt levels, increase of inflammatory mediators, hypercortisolism and/or somatotropic dysfunction [32,35]. The reversibility of sarcopenia in a model of fatty liver mediated by supplementation of GH or IGF-1 has not yet been demonstrated.

Based on this data, the hypothesis of the study is that somatotropic dysfunction is involved in pathogenesis at the early stages of NAFLD development inducing insulin resistance, liver steatosis, adipocyte dysfunction and sarcopenia. Supplementation of the somatotropic axis can be associated with significant beneficial effects, at the liver and systemic levels. However, supplementation with GH and IGF-1 can have dissimilar effects, particularly in modulating insulin resistance, adipose tissue function and lipid metabolism in the liver. The aim of this study was to analyse and compare the effects of GH and IGF-1 supplementation in liver, adipokines and muscular tissue in an NAFLD experimental model that resembles features of early NAFLD stages including obesity and insulin resistance.

## 2. Results

### 2.1. Metabolic Parameters: Growth Hormone Increased Serum Insulin and Induced Hepatomegaly

**Figure 1:**
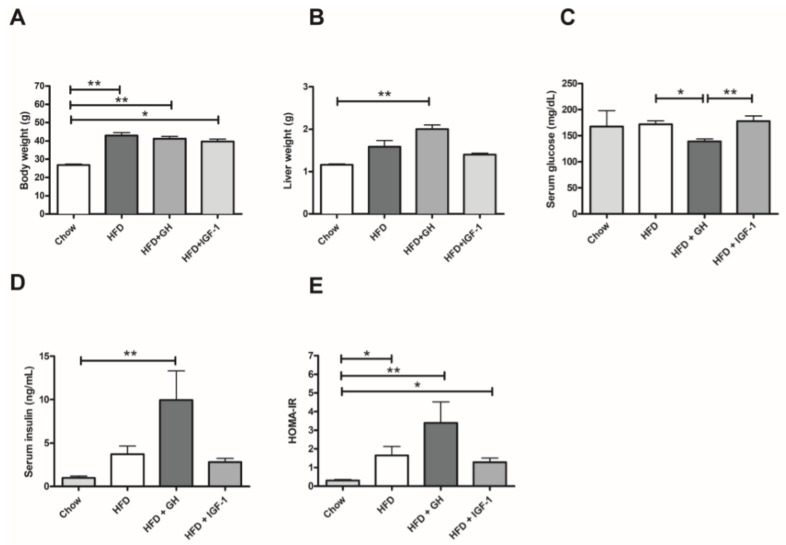
Metabolic effects of intervention according to study groups: (**A**) Body weight; (**B**) Liver weight; (**C**) Serum Glucose; (**D**) Serum Insulin and (**E**) Homeostasis Model Assessment of Insulin resistance (HOMA-IR). High fat diet (HFD) induced a significant increase in body weight and HOMA-IR. HFD induced hepatomegaly and a significant increase in serum insulin. * *p* < 0.05; ** *p* < 0.01.

**Figure 2:**
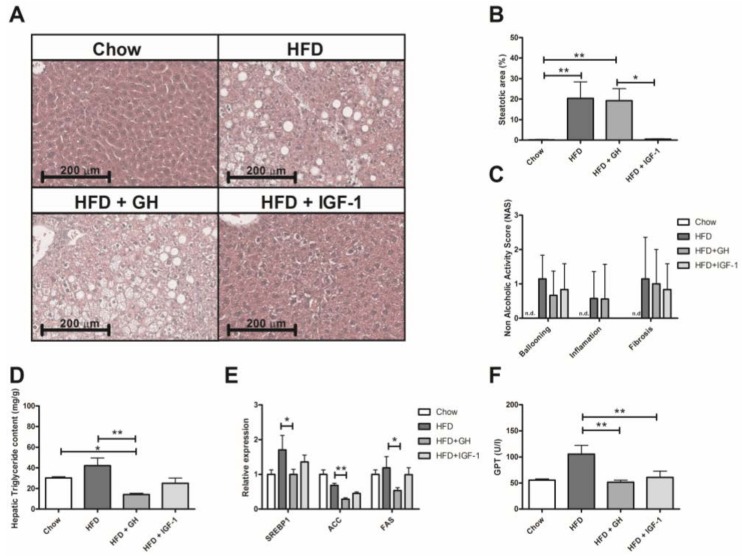
Effects of somatotropic hormone supplementation in liver. (**A**) Representative histological images of livers from experimental groups after 22 weeks of feeding with either chow, high fat diet (HFD) and HFD supplemented with growth hormone GH or insulin-like growth factor-1 (IGF-1). (**B**) Quantification of liver steatosis by histology in percentage of steatotic hepatocytes in experimental groups. No significant increase in steatosis can be observed in HFD + IGF-1 group compared to Chow. (**C**) Histology Score. No significant changes were observed in liver inflammation, ballooning and fibrosis in histology. (**D**) Hepatic triglycerides content according to experimental groups. A significant reduction of triglycerides content was observed in HFD + GH group. (**E**) Effects of hormone supplementation in lipogenic gene expression. A significant reduction was observed in HFD + GH group compared to HFD. (**F**) Serum ALT levels according to experimental groups. A significant reduction was observed in HFD + GH and HFD + IGF-1 groups compared to HFD. * *p* < 0.05; ** *p* < 0.01.

Mice fed with a HFD for 12 weeks presented a phenotype of obesity with steatosis. The 3 mice groups exposed to HFD significantly gained weight (25–30%, *p* < 0.01) regarding the control group (Figure 1A). No significant difference was observed between the supplemented group with GH (HFD + GH) or IGF-1 (HFD + IGF-1) compared with HFD group. In addition, HFD + GH mice supplemented with GH had significantly greater liver weight (~80% increase, *p* < 0.01) than chow group (Figure 1B). HFD and HFD + IGF-1 groups did not show significant differences in liver size compared to the control group. Serum glucose, after 6 hours’ fast, did not present differences between the control and HFD groups (Figure 2C). However, HFD + GH exhibited minor serum glucose levels compared to HFD (171.7 ± 6.53 vs. 139.2 ± 4.61 mg/dL, *p* < 0.05) and HFD + IGF-1 (177.9 ± 10 mg/dL, *p* < 0.01). All groups that received HFD presented higher serum insulin levels compared to the control, but only HFD + GH presented a significant difference compared to the control group (0.98 ± 0.22 vs. 10 ± 3.35 µIU/mL, *p* < 0.01) (Figure 1D). The Homeostasis Model Assessment of Insulin resistance (HOMA-IR) index increased significantly in the groups fed with HFD compared to the chow group (*p* = 0.004). No difference in this index was observed between the HFD mice and the HFD supplemented with GH or IGF-1 (Figure 1E). Together, these results indicate that GH increased serum insulin and induced hepatomegaly in mice fed with HFD.

### 2.2. Somatotropic Hormone Supplementation Was Associated with Improvement in Liver Lipid Content and Serum Transaminases

HE sections of every study group were analysed by a blind pathologist (Figure 2A). The groups fed with HFD and HFD + GH presented a significantly higher steatosis NAS score compared to the chow group (HFD: median 0% p25–75% = 0–0.25; HFD + GH: median 12.5% p25–75% = 2.5–37.5 and chow group: median 0% p25–75% = 0–0.5, *p* < 0.01), unlike the HFD + IGF-1 group, which showed no difference compared to the control (median 1%, p25–75% = 0–1, *p* = 0.4) (Figure 2B). No flashpoints, ballooning or significant fibrosis elements were found in any of the groups (Figure 2C). In the specific analysis for liver triglyceride content, the HFD + GH group presented significantly lower levels compared to the chow and HFD groups (HFD + GH: 14.3 ± 1.1; Chow: 30.18 ± 1.3 and HFD: 42.1 ± 7.4 mg/g; *p* < 0.05 and *p* < 0.01, respectively) (Figure 2D). In the HFD + IGF1 group, liver triglycerides were similar to the chow group (25.1 ± 5.1 mg/g). To better explore this finding, the expression of hepatic lipogenesis enzymes (sterol regulatory element-binding protein 1, SREBP1; Acetyl-CoA carboxylase, ACC; and fatty acid synthase, FAS) were evaluated, and were found to be decreased in the HFD + GH compared to the HFD group (*p* = 0.05; *p* = 0.002 and *p* = 0.02 respectively). No difference was observed in the other groups (Figure 2E).

The activity of serum alanine aminotransferase (ALT) doubled in mice fed with HFD compared to a chow diet. Supplementation of HFD mice with either GH or IGF-1 significantly reduced ALT levels compared to HFD (HFD: 105.4 ± 16.9; HFD + GH: 51.6 ± 3.8 and HFD + IGF-1: 60.7 ± 12 IU/L; *p* < 0.01 for both) (Figure 2F). No differences regarding TNFα or MCP1 expression were observed between groups fed with HFD (Supplementary Figure S1). These results suggest that somatotropic axis supplementation has positive impacts on liver transaminases and lipid metabolism, reducing lipogenesis (GH) and liver steatosis (IGF-1).

### 2.3. Sarcopenia Was Reverted by Hormone Supplementation and Growth Hormone Induced Significant Reduction in Serum Adipokines

**Figure 3:**
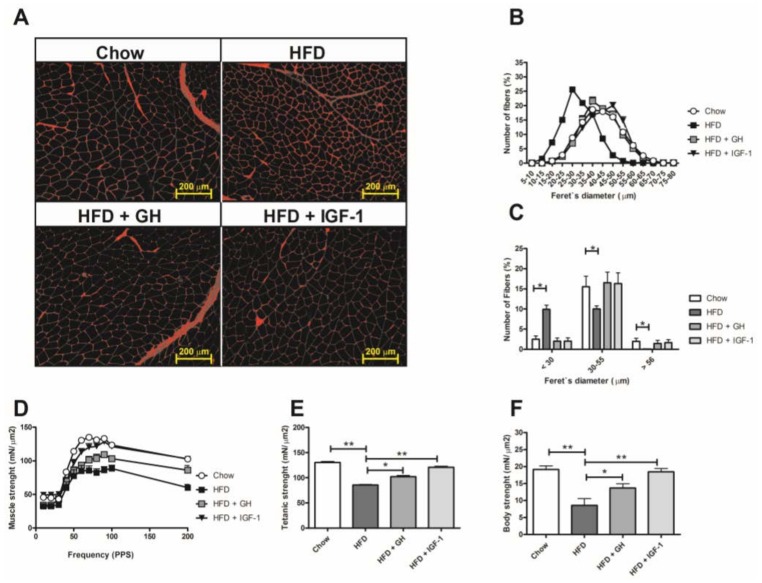
High fat diet induced sarcopenia that was fully reverted by somatotropic hormone supplementation. (**A**) Muscle histological sections with wheat germ agglutinin (WGA) fluorescence staining, (**B**) muscle fibre diameter distribution as determined by measure of the minimal feret’s diameter, (**C**) analysis of three muscle fibre size ranges. Thick (>56 Lm), medium size (30–55 Lm), and thin (<30 Lm) muscle fibres are shown. (**D**,**E**) electrophysiological analysis of TA muscle strength from both groups including twitch and tetanus contractions. (**F**) Body strength in vivo. * *p* < 0.05; ** *p* < 0.01; N.D., not detectable.

Using a fluorescent molecule (WGA) to delimit sarcolemme and measure muscular fibres diameters (Figure 3A), there was a higher percentage of thin fibres (<30 µm) and a lower percentage of thick fibres (>56 µm) in the HFD group compared to control group, evidenced by a displacement to the left of the normal curve (Figure 3B,C). Regarding muscle function, mice fed with HFD had significantly less strength in vivo and lower contractile response in electrophysiology analysis compared to the chow group (Figure 3). HFD + GH and HFD + IGF-1 evidenced a significant reversion of sarcopenia at muscle structure level (fibre diameter) (Figure 3A–C) and significantly greater strength measurements than HFD assessed by ex vivo measurements through electromyography (Figure 3D,E) and in vivo assays (Figure 3F). Interestingly, mice supplemented with IGF-1 presented ranges of strength similar to the control group with chow diet (Figure 3D,F).

**Figure 4:**
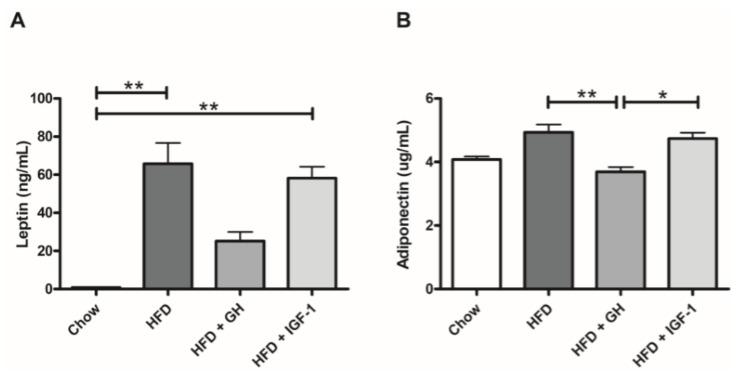
Serum adipokines levels: (**A**) Leptin and (**B**) adiponectin. Growth hormone supplementation attenuated high fat diet (HFD) associated increase of leptin serum levels, and reduced serum adiponectin levels compared to HFD and HFD + IGF-1. * *p* < 0.05; ** *p* < 0.01.

A significant increase in the serum levels of leptin was observed in HFD and HFD + IGF-1 groups compared to the chow group (HFD: 65.8 ± 10.9; HFD + IGF-1: 58.2 ± 6 and Chow: 0.9 ± 0.1 ng/mL; *p* < 0.01 for both), which was not observed in the HFD + GH group (25.2 ± 4.7 ng/mL). In addition, the HFD + GH group demonstrated a significant decrease in serum adiponectin levels compared to the HFD and HFD + IGF-1 groups (HFD: 4.9 ± 0.2; HFD + GH: 3.7 ± 0.2 and HFD + IGF-1: 4.7 ± 0.2; *p* < 0.01 and *p* < 0.05, respectively) (Figure 4).

## 3. Discussion

The present study evaluated supplementation of the somatotropic axis with IGF-1 and GH in an NAFLD experimental model. IGF-1 treatment reduced histological hepatic steatosis, serum ALT and reversed the development of sarcopenia associated with NAFLD. GH treatment presented similar effects regarding ALT and sarcopenia reduction; however, it was associated with hepatomegaly, hyperinsulinemia, the reduction of serum levels of leptin and adiponectin and no significant steatosis reduction in histology assessment. These results suggest somatotropic axis supplementation has beneficial effects in our model, with a safer profile for direct IGF-1 supplementation.

This experimental model achieved the development of the expected phenotype (obesity and IR). None of the supplemented groups showed a reversal of this phenotype regarding significant weight changes or HOMA-IR induced by the HFD. Indeed, the HFD + GH group presented increased serum insulin. Mice supplemented with GH presented also a significant glycaemia reduction compared to the HFD and HFD + IGF-1 groups. This finding suggests a direct effect of GH supplementation on serum insulin levels. A previous study (in humans) of the effects of GH supplementation on insulin resistance found that it is dose-dependent and varies depending on the metabolic characteristics of treated subjects. Most of the studies using high dose GH supplementation were associated with at least a short term (<6 months) increase in serum insulin and insulin resistance [36]. In addition, GH has been described to directly increase the synthesis of IGF-1 in pancreatic islets and induce a direct increase in insulin secretion through the preservation of beta cells of the islets and to promote insulin synthesis and sensitization of these cells to serum glucose changes [36,37]. IGF-1 reduces GH expression at hypophysis, reducing the hyperinsulinemic effect of GH. In our model, direct systemic IGF-1 supplementation was not associated with hyperinsulinemia. 

Regarding the liver effects of somatotropic axis supplementation, three facts are relevant to be highlighted. First, the enlargement of liver size observed in the HFD + GH group. GH induced a hypertrophic and hyperplastic effect at the hepatocellular level that has been previously described [38]. This effect was not observed in the group supplemented with IGF-1. This feature might be relevant, considering the potential carcinogenic effect of GH supplementation. Secondly, the group supplemented with GH showed lower triglyceride content in the liver compared to the HFD and control groups, probably secondary to the inhibition of hepatic lipogenesis as demonstrated by a reduction in lipogenic enzyme (SREBP-1, ACC and FAS) expression in liver samples (Figure 3E). This effect was previously reported by Córdoba-Chacón et al. [39]. However, the histological analysis revealed that GH did not reduce liver steatosis induced by HFD, suggesting that steatosis could be determined by the accumulation of lipids different from triglycerides (diacyglicerol, desmosterol, cholesterol, ceramides). In the HFD + IGF1 group, liver triglycerides were similar compared to control group levels, and no significant changes in the expression of lipogenic enzymes were observed. IGF-1 supplementation reverted liver steatosis induced by HFD at histology analysis. Recently, Nishizawa et al. demonstrated that IGF-1 improved mitochondrial function and reduced liver steatosis in a model of mice with a methionine- and choline-deficient diet [29]. This finding represents a potential pathway involved in IGF-1-mediated steatosis: autophagy and lipidic oxidation at the mitochondrial level. Another significant finding was the normalization of ALT in the HFD groups supplemented with GH and IGF-1. This finding was not associated with changes in the hepatic expression of inflammatory markers (MCP1; TNFα) (Supplementary Figure S1). The studied model (HFD) is centred around the metabolic changes observed in NAFLD rather than important inflammatory phenomena. The changes in AST levels could be related to lower cellular stress (e.g., mitochondrial and endoplasmic reticulum), and correspondingly, lower hepatocellular apoptosis and necrosis, which is in line with the report by Nishizawa et al.

The skeletal muscle analysis revealed that HFD induced a significant reduction in muscle strength and fibre diameter compatible with sarcopenia as previously reported by Abrigo et al. [40]. Supplementation with GH and IGF-1 reversed this phenomenon: muscular fibres regained their size (diameter similar to the chow group) and muscle function in vivo and ex vivo was normalized. Even though supplementation with IGF-1/GH is associated with an anabolic effect expected at the muscular level, only a minor response has been raised in other aetiologies of sarcopenia such as ageing-induced sarcopenia [41,42,43,44]. Interestingly, despite using low doses of IGF-1 in supplementation, it demonstrated an equal, or even slightly higher, effect to supplementation with GH. Recently, Consitt L et al. described that mice chronically exposed to supraphysiologic levels of GH (four times over normal serum value) inhibit muscular hypertrophy through a raise in myostatin levels, expression of muscle RING-finger protein-1 (MuRF1) and phosphorylation of the serine group of insulin receptor substrate 1 (IRS1) [45]. These findings are in line with the hypothesis of a potential resistance to the direct effect of GH at a muscular level that might not be observed with supplementation with IGF-1. This observation, added to the safer metabolic profile of IGF-1, with a lower liver hypertrophic effect and a higher histological response regarding steatosis, suggests that direct supplementation with IGF-1 presents a more attractive therapeutic profile than supplementation with GH. This finding is relevant considering the currently ongoing randomized controlled trials with growth hormone and growth hormone releasing hormone analogue (tesamorelin) in subjects with fatty livers (NCT02217345, NCT03375788).

Finally, supplementation with GH was associated with a reduction in serum levels of leptin and adiponectin, which was not observed in the group supplemented with IGF-1. The reduction of adipokines induced by GH could be explained by adipose tissue reduction, which has previously been demonstrated to be associated with supraphysiological doses of GH (higher than 5 ug/g/d) [25]. Although this effect could be considered beneficial, in individuals with acromegaly, this effect is associated with ectopic deposits of fat and insulin resistance [27]. GH has also been reported to induce a direct fat mass independent effect in leptin and adiponectin serum levels [46,47]. In our model, this effect was not reproduced by direct IGF-1 supplementation.

In conclusion, the supplementation of the somatotropic axis in a murine model of HGNA normalized aminotransferases, reversed sarcopenia and reduced hepatic triglycerides (mainly GH supplementation) and steatosis (IGF-1 supplementation). Unlike GH, IGF-1 supplementation was not associated with potential adverse effects like hepatic volume increase and hyperinsulinemia. The incorporation of somatotropic axis hormone supplementation, particularly low dose IGF-1, as a pharmacological therapy in patients with the triad of NAFLD, insulin-resistance and sarcopenia could be promising. However, studies with supplementation for longer periods of time are required to properly evaluate the safety profile and the chronic effect of intervention.

## 4. Materials and Methods

**Figure 5:**
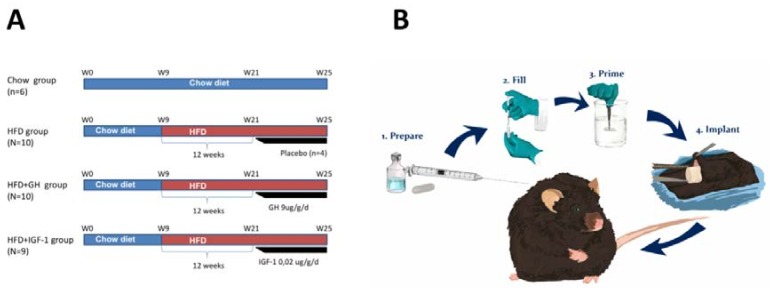
(**A**) Schematic representation of experimental approach on each of the study groups. (**B**) Implantation of osmotic pumps. Osmotic pumps were implanted subcutaneously in C56BL6 mice after 12 weeks of high fat diet for continuous subcutaneous infusion of placebo, growth hormone (GH) or insulin growth factor type 1 (IGF-1) respectively. HFD: High fat diet.

**Animals and diet:** male C57BL6 mice from the Animal Facility of Department of Gastroenterology, Pontificia Universidad Católica de Chile, were used for the experimental studies, following the animal use and care protocols revised and approved by the committee of animal care and well-being of the university’s School of Medicine (CEBA 14-047, 10 June 2014). The animals were kept in conditions of controlled temperature and light, in polycarbonate cages. The animals received water ad libitum and a balanced diet before initiating the study protocol (Prolab 3000, Purina, PMI Feeds Inc., St. Louis, MO, USA). At the age of 9 weeks, the mice were fed with a high fat diet (HFD) (Protein 20 kcal%, Carbohydrates 20 kcal% and fat 60 kcal%, 5.24 kcal/g, Research diets Inc. D12492 New Brunswick, NJ, USA) for 12 weeks. Animals with a weight increase less than 30% were excluded from the study. Later, the animals were divided into 3 groups and received for an additional 4 weeks: (1) HFD; (2) HFD, supplemented with GH 9 µg/g/day by a subcutaneous osmotic pump (Figure 5) (HFD + GH) (ALZET^®^ Osmotic Pumps, Cupertino, CA, USA); and (3) supplemented with IGF-1 0.02 µg/g/day by an ALZET^®^ pump (HFD + IGF-1) (Figure 5A,B). At the same time, 6 mice were kept for the same period of time, fed a chow diet as the control group. A subgroup (*N* = 4) were fed HFD and received placebo (sham) ALZET pumps. The euthanasia of the mice was carried out by bleed-out under anaesthesia and samples of serum, liver, muscle and adipose tissue were collected and stored at −80 °C until analysis.

**Biochemical determinations:** In serum: Alanine aminotransferase (ALT) was measured with a Kovalent kit (Río de Janeiro, Brazil). Insulin was measured using a Millipore kit (Merck, Billerica, MA, USA). Glucose serum levels were determined with a capillary hemoglucotest (One Touch, Johnson and Johnson Medical Devices and Diagnostics Group—Latin American) after a 6 h fast. HOMA-IR (Homeostasis Model Assessment of Insulin resistance) was calculated according to the formula ((fasting serum glucose mg/dL × fasting serum insulin ng/mL)/405). In the liver, the levels of triglycerides (TGL) were measured with Sigma reactive (St. Louis, MO, USA) in 50–80 mg of homogenated tissue in 1.5 mL of a mixture of chloroform: methanol (2:1 *v*/*v*), followed by a Folch extraction.

**Histological analysis:** Liver sections from the right lobe of all mouse livers were routinely fixed in 10% formalin and embedded in paraffin. Then 4 μm of tissue sections were stained with hematoxylin/eosin (HE). A researcher, blind to the experimental groups (pathologist), evaluated the cuts and assigned a score of steatosis, inflammation and fibrosis. Fibrosis was evaluated with picrosirius red solution 0.1%.

**Quantitative PCR in Real Time:** Total *ribonucleic acid* (*RNA*) was isolated from the liver tissue using a Speed Vacuum Total RNA Isolation System (Promega Corporation, Madison, WI, USA). The RNA was quantified by absorption at 260 nm in a spectrophotometer Nanodrop ND-1000 (Thermo Fisher Scientific, Wilmington, DE, USA). Splitters designed by Primer Express software (Applied Biosystems, Lincoln, CA, USA) were used for the specific studied genes. A detailed table of primers used for these assays is included in the supplementary material. As a control, mRNA 18S was used and the relative quantities of mRNA were calculated using the method of the Threshold Cycles. The expression of markers of inflammation (tumour necrosis factor α (TNFα) and monocyte chemoattractant protein-1 (MCP-1)) was evaluated in the livers.

**Muscular Study:** The muscular strength in vivo was evaluated using a chain technique [48]. This technique is detailed in the supplementary material. The muscle strength ex vivo was evaluated in the right gastrocnemius by electro-stimulation at different frequencies immediately following the sacrifice of the mice. The optimal muscular strength and voltage of stimulation were determined by micromanipulation of the muscular force produced (maximum isometric contraction). The tetanus force was plateau determined between force-frequency, after successive stimulations from 1 to 200 Hz for 450 ms, with a 2-min rest between each stimulus. After determining the isometric contractile properties, muscles were subjected to 3 repeated tetanus stimulations. The optimal muscular force was stimulated for 450 ms every 5 s. Once the functional muscular test had been performed, the tendon and any other non-muscular tissue were eliminated to determine the muscular mass and calculate the specific net force (mN/mm^2^). The diameter of muscular fibres was measured in wheat germ agglutinine (WGA) samples of muscle using the method described by Cabrera D et al. [49]. Briefly, fibre size was determined using the ImageJ software on five randomly captured images at 400× magnification for each muscle section stained with WGA (Invitrogen™ Molecular Probes™, CA, USA). WGA is a lectin that binds to fibre sarcolemma glycoproteins on the basement membrane and effectively outlines the fibre periphery to allow measurement of fibre size. Fibres were manually selected and the minimal Feret’s diameter of each fibre was determined using digital image analysis software (ImageJ, NIH, Bethesda, MD, USA) [50].

**Statistical analysis:** The results are expressed as mean ± SE, using the two-tailed couplets Student’s T-test to compare the differences between groups. For non-parametric variables, the Mann-Whitney *U*-test or Kruskal-Wallis test were used. The values were considered significantly different when the *p* value was equal, or inferior, to 0.05. The ANOVA test was used for multiple group comparisons. In the post-hoc analysis, Dunn were used to correct multiple comparisons. All the data were analysed and plotted with the program GraphPad 6 Prism (GraphPad Software, Inc., La Jolla, CA, USA).

**Weightlifting Strength Test:** The muscle strength of the mice was measured through a weightlifting test, as previously described [48]. Briefly, the apparatus consisted of a series of increasingly long chain links (and weight) attached to a ball of tangled fine wire. Before performing the test, and prior to treatments, the mice were trained once per day for two weeks. To perform the test, the mouse grasped the different weights with its forepaws and a score was assigned. The final score was calculated as the summation of the product between the link weight and the time the weight was held. The average of three measures from each mouse was normalized against body weight [40].

## Supplementary Materials

Supplementary materials can be found at http://www.mdpi.com/1422-0067/19/5/1339/s1.

## Abbreviations

NAFLD	non–alcoholic fatty liver disease
GH	Growth Hormone
IGF-1	insulin-like growth factor 1
HFD	High Fat Diet
ALT	Alanine aminotransferase
CSA	Cross sectional area
NASH	Non-alcoholic steatohepatitis
IR	Insulin resistance
T2DM	Type 2 diabetes mellitus
DXA	Dual energy X-ray absorptiometry
GHRH	Growth Hormone Releasing Hormone
HOMA-IR	Homeostasis Model Assessment of Insulin resistance
HTC	Hepatic triglyceride content
HE	Hematoxylin/eosin
N.D.	Not detected
MCP-1	Monocyte chemoattractant protein-1
RT-PCR	Real Time-Polimerase Chain Reaction
SREBP1	Sterol regulatory element-binding protein 1
ACC	Acetyl-CoA carboxylase
FAS	Tumor Necrosis Factor-α
usPCR:	Ultrasensitive C Reactive Protein
VAT	Visceral Adipose Tissue
WGA	Wheat germ agglutinin
