# Supplementary material for: Somatotropic Axis Dysfunction in Non-Alcoholic Fatty Liver Disease: Beneficial Hepatic and Systemic Effects of Hormone Supplementation
Source: Int J Mol Sci. 2018 May 2;19(5):1339. doi: 10.3390/ijms19051339 (PMC5983806; doi:10.3390/ijms19051339)
Supplement: Supplementary file 1 [file ijms-19-01339-s001.zip › ijms-282561-supplementary.pdf]

Supplementary Materials:

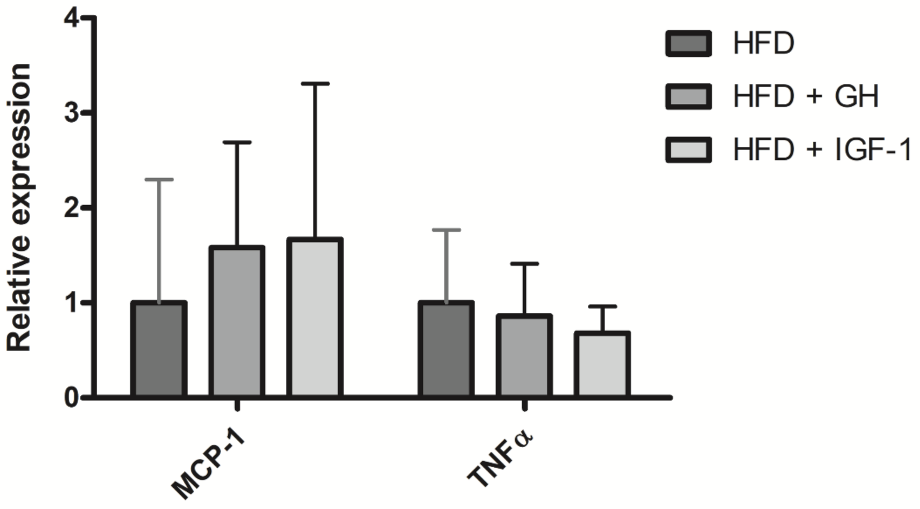

Supplementary figure 1. No significant difference was observed in liver inflammatory mediator expression (monocyte chemotactic protein 1, MCP-1; tumor necrosis factor α, TNFa)

Supplementary Table 1. Summary of qPCR primers included in the study

Item Data	Gene	Species	Forward (5'-->3')	Reverse (5'-->3')	NCBI Access Nº	PCR product (bp)
m SREBP-1c	sterol regulatory element binding transcription factor 1 (SREBF1c)	Mus Musculus	GGA GCC ATG GAT TGC ACA TT	GGC CCG GGA AGT CAC TGT	XM_006532716.1	70
m Acaca	acetyl-Coenzyme A carboxylase alpha (Acaca)	Mus Musculus	TGACAGACTGATCGCAGAGAAAG	TGGAGAGCCCCACACACA	NM_133360.2 (transcript variant)	75
m Fasn	fatty acid synthase (Fasn)	Mus Musculus	GCTGCGGAAACTTCAGGAAAT	AGAGACGTGTCACTCCTGGACTT	NM_007988.3	84
m Scd1	stearoyl-Coenzyme A desaturase 1 (Scd1)	Mus Musculus	CCGGAGACCCCTTAGATCGA	TAGCCTGTAAAAGATTTCTGCAAACC	NM_009127.4	89
TNF-A	tumor necrosis factor (Tnf)	Mus Musculus	CCC TCA CAC TCA GAT CAT CTT CT	GCT ACG ACG TGG GCT ACA G	NM_013693.3	61
MCP-1	chemokine (C-C motif) ligand 2 (Ccl2)	Mus Musculus	TTAAAAACCTGGATCGGAACCAA	GCATTAGCTTCAGATTTACGGGT	NM_011333.3	121
